# Successful calcium modification of a large calcified nodule using shockwave intravascular lithotripsy in the setting of acute coronary syndrome: a case report

**DOI:** 10.1093/ehjcr/ytae517

**Published:** 2024-09-19

**Authors:** Arif A Al Nooryani, George Sianos, Nagwa Abdelrahman

**Affiliations:** Cardiovascular Department, Al Qassimi Hospital, Sharjah, 3500, UAE; Cardiovascular Department, Al Qassimi Hospital, Sharjah, 3500, UAE; Cardiovascular Department, Al Qassimi Hospital, Sharjah, 3500, UAE; Cardiovascular Department, Faculty of Medicine, Assiut University, Assiut, 71515, Egypt

**Keywords:** Calcium nodule, Case report, Intravascular lithotripsy, Intravascular ultrasound, Rotablation

## Abstract

**Background:**

Calcified nodules are associated with suboptimal preparation before stenting due to challenging crossing and unsuccessful pre-dilation and calcium cracking with conventional balloons. In this scenario, we report the use of shockwave intravascular lithotripsy for the successful lesion preparation of an undilatable and challenging calcified nodule in a patient presenting with ACS.

**Case summary:**

We report a case of a 79-year-old male patient presented with non-ST elevation myocardial infarction. Coronary angiography revealed 90% stenosis in the proximal segment of the right coronary artery, with a hazy area of inhomogeneous contrast. Intravascular ultrasound (IVUS) imaging identified a large eccentric calcified nodule, with a minimum luminal area (MLA) of 4.18 mm^2^. Rotablation was done with a ROTAPRO Atherectomy System, post-rotablation IVUS showed no plaque modification. Intravascular lithotripsy (IVL) was performed with the emission of 50 pulses. Post-IVL, IVUS showed that the calcium nodule was successfully cracked with increased MLA to 6.8 mm^2^. The lesion was pre-dilated with a cutting balloon and stented using a SYNERGY MEGATRON stent and post-dilated with a non-compliant balloon with good final angiographic result and TIMI Grade 3 flow. Post-stenting IVUS confirmed optimal stent apposition and expansion with an MLA of 11.9 mm^2^.

**Discussion:**

In severely calcified lesions, like calcified nodules, lesion preparation before stenting is pivotal for optimal long-term outcomes. As demonstrated in this case, IVL can be used safely in the setting of ACS not only to treat superficial and deep calcium layers but also to crack a large, calcified nodule, after failure of rotablation.

**Learning points**

- Intravascular imaging modalities such as intravascular ultrasound and optical coherence tomography should be used to assess the anatomic characteristics of severely calcified lesions.
- Intravascular lithotripsy can be used safely to crack a large, calcified nodule, in case of rotablation failure.

## Introduction

Calcified nodule is the least common cause of acute coronary syndrome (ACS).^1^ It is defined as a lesion with multiple erosive and dense calcific nodules which disrupt the overlying fibrous cap prompting thrombus formation.^2^ Emerging evidence associate calcified nodules with increased adverse clinical outcomes in the setting of ACS, including sudden cardiac death.^3^ Yet, its exact pathophysiology remains an enigma. Lesions with high calcific burden are associated with suboptimal preparation before stenting due to challenging crossing and unsuccessful pre-dilation and calcium crack with conventional balloons. In this scenario, we report the use of intravascular lithotripsy (IVL) for the successful lesion preparation of an undilatable and challenging calcified nodule in a patient presenting with ACS.

## Summary figure

**Figure:**
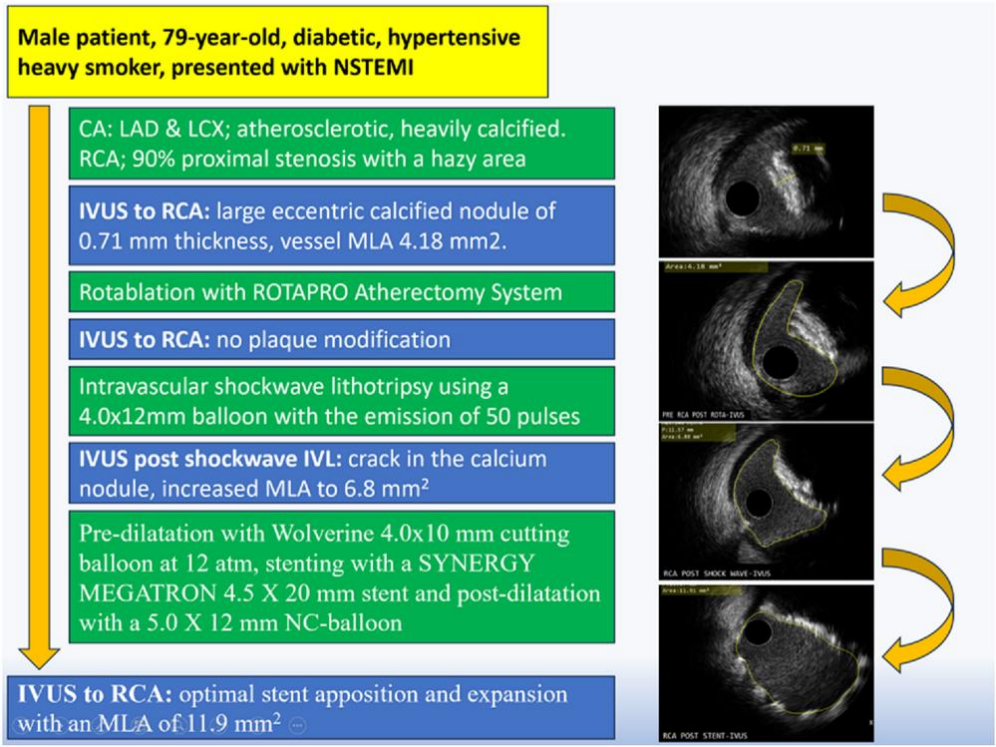


## Case presentation

A 79-year-old male presented in the emergency department with typical chest pain which lasted for 15 min. At presentation, the patient was afebrile, haemodynamically stable with normal oxygen saturation at room air. Physical examination revealed no murmurs and normal breath sounds. The electrocardiogram (ECG) showed sinus rhythm without ischaemic-appearing changes. Non-ST elevation myocardial infarction (NSTEMI) was diagnosed through the combination of symptoms and elevated high-sensitivity troponin levels. He was loaded with aspirin and ticagrelor, 5000 units of unfractionated heparin intravenously, and underwent coronary angiography (CA).

The patient had well-controlled diabetes mellitus type 2, hypertension, and 60 pack years of smoking. He had previously established coronary artery disease (CAD), which consisted of percutaneous coronary intervention (PCI) in the distal right coronary artery (RCA). He had impaired left ventricular (LV) systolic function with an ejection fraction of 40% and moderate chronic kidney disease with baseline serum creatinine of 124 umol/L and glomerular filtration rate of 47.4 mL/min.

**Figure 1:**
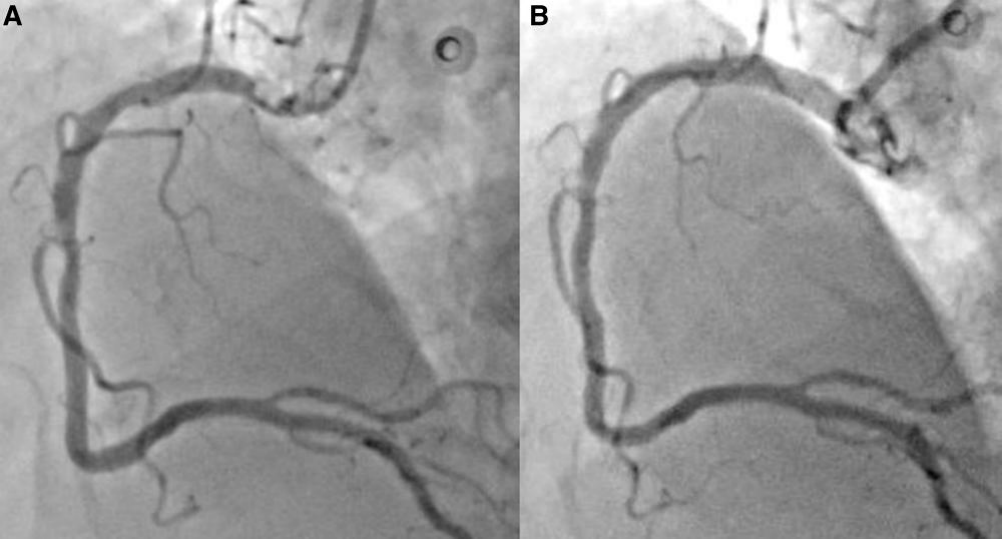
*(A)* Right coronary artery angiogram showing calcium nodule as a hazy area in the proximal segment with severe luminal narrowing. *(B)* Post-percutaneous coronary intevention to ostial right coronary artery showing good angiographic result with proper stent expansion.

**Figure 2:**
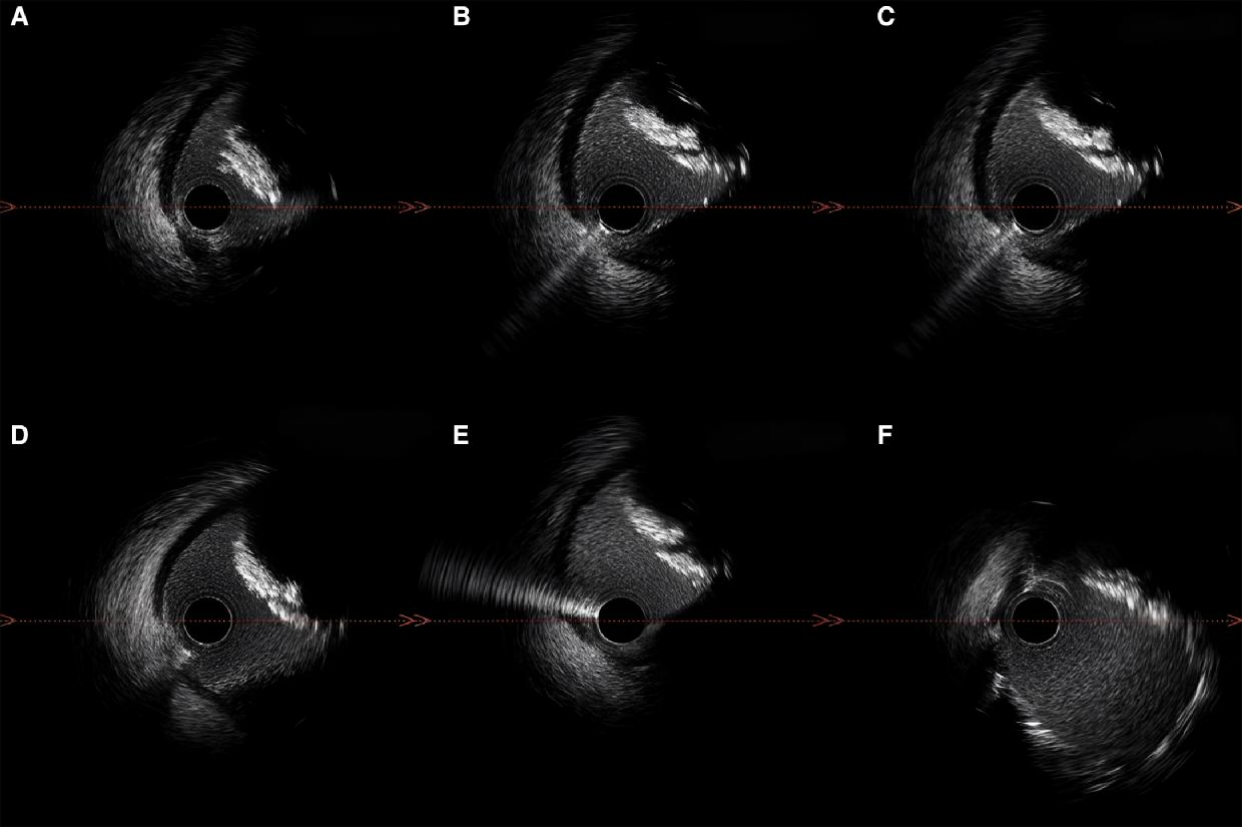
Intravascular ultrasound images of the right coronary artery showing eccentric calcified nodule without any cracks post-rotablation *(A)*, then cracked calcium nodule at multiple levels following intravascular lithotripsy *(B–E)*, and final imaging post-stenting showing good stent expansion and proper apposition *(F)*.

The CA was performed via radial approach and revealed severe calcification of the left anterior descending artery (LAD) and the left circumflex artery (LCx) with minimal luminal irregularities. The RCA was severely diseased with 90% proximal stenosis. During contrast injection, a hazy area with inhomogeneous contrast staining in the proximal RCA made the differentiation of thrombus from calcium content challenging *(Figure 1A)*. The use of intravascular ultrasound (IVUS) identified a large eccentric calcified nodule (CN) of 0.71 mm thickness, with a calculated minimum luminal area (MLA) of 4.18 mm^2^ (*Figure 2A*).

First, a Terumo Runthrough NS Extra Floppy Guide Wire was unsuccessfully used to access the distal part of RCA. A Finecross MG Coronary Micro-Guide Catheter integrated crossability and guidewire support and provided easy passage of the Super-Support RotaWire which was positioned at the distal part of the target vessel. A ROTAPRO Atherectomy System with a burr size of 1.5 mm was advanced over the RotaWire. No resistance was met during the short individual runs of rotablation. Even after multiple rotational atherectomies (RAs), post-RA IVUS pullback showed rotablation therapy failure with no plaque modification highlighting the high calcific burden of the target lesion. Accordingly, shockwave IVL was performed by introducing an IVL balloon 4.0 × 12 mm to the site of the nodular calcification; the balloon was dilated up to 4 atm followed by the emission of pulsatile mechanical energy of 50 pulses. Post-IVL, IVUS showed that the CN was successfully cracked with increased MLA to 6.8 mm^2^ (*Figure 2B–E*). Then, the lesion was pre-dilated with a cutting balloon (Wolverine) 4.0 × 10 mm at 12 atm, and post-dilation IVUS verified optimal lesion preparation. A 4.5×20 mm SYNERGY MEGATRON BP Stent was deployed and post-dilated with a 5.0×12 mm non-compliant balloon with good final angiographic result and TIMI Grade 3 flow (*Figure 1B*). Post-stenting IVUS confirmed optimal stent apposition and expansion with an MLA of 11.9 mm^2^ (*Figure 2F*). A total of 120 mL of Iso osmolar iodixanol (Visipaque; GE Healthcare, Little Chalfont, Buckinghamshire, UK) was used during the procedure. Post-procedural course was uneventful apart from mild increase in serum creatinine up to 150 umol/L (21%), corrected by intravenous hydration. High-sensitivity troponin-I level declined from 11 515.1 ng/L pre-procedure to 1525.4 ng/L on fourth day post-PCI (normal range 0.0–60.4 ng/L).

## Discussion

In severely calcified lesions, like calcified nodules, lesion preparation before stenting is pivotal for optimal long-term outcomes. Advanced intravascular imaging modalities (IVUS and optical coherence tomography) should be used to assess the anatomic characteristics of severely calcified lesions. Rotational atherectomies can be performed in superficial calcified lesions with increased calcium thickness and calcium length, while IVL has a preferential effect on the deep calcium.^4^ Numerous prospective and retrospective registries have assessed the efficacy and safety of coronary IVL.^5–8^ The largest study, ‘Disrupt CAD III With the Shockwave Coronary IVL System’, involved 431 patients with stable, unstable, or silent ischaemia and demonstrated the safety and effectiveness of IVL for calcified lesion preparation prior to stenting.^8^ While excluded from Disrupt CAD III study, NSTEMI patients presented 40% (169 patients) of the REPLICA-EPIC18 study population. This study revealed a non-significant increase in 30-day major adverse cardiac events in ACS patients, although their angiographic success rates were comparable to those of chronic stable patients.^9^ However, since IVL is a balloon-based technique, uncrossable lesions pose a main limitation for its use. In our case, the lesion was crossed without any resistance with IVUS, but RA did not disrupt the characteristics of the calcified nodule. After failed RA, IVL successfully cracked this calcified nodule, enabling the complete semi-compliant balloon dilatation to reference vessel size and full expansion of a MEGATRON stent.

## Conclusion

Calcified nodule constitutes a rare cause of ACS. This case highlights the importance of dedicated devices for lesion preparation in undilatable highly calcific lesions. As demonstrated in this case, IVL can be used safely in the setting of ACS not only to treat superficial and deep layer calcium but also to crack a large, calcified nodule, after failure of RA.

## Lead author biography


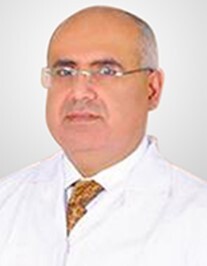


Dr Al Nooryani is a consultant interventional cardiologist; he is currently the CEO of Al Qassimi Hospital, Sharjah, UAE. He is a professor of cardiology at the University of Sharjah. He received his Bachelor’s Degree of Medicine from ULM, Germany, followed by German Board of Internal Medicine and Cardiology certifications.

## Supplementary Material

ytae517_Supplementary_Data

## Data Availability

The data underlying this article can be provided upon request.

## Notes

**Consent:** The authors confirm that written consent for submission and publication of this case report has been obtained from the patient in line with COPE guidelines.

**Funding:** The case report didn't receive any specific grant from any funding agencies.
